# Measurement of Bed Turning and Comparison with Age, Gender, and Body Mass Index in a Healthy Population: Application of a Novel Mobility Detection System

**DOI:** 10.1155/2014/819615

**Published:** 2014-04-29

**Authors:** Shang-Lin Chiang, Chia-Huei Lin, Shin-Tsu Chang, Chueh-Ho Lin, Po-Yin Chen, Wen-Hsu Sung, Shun-Hwa Wei

**Affiliations:** ^1^Department of Physical Therapy and Assistive Technology, National Yang Ming University, No. 155, Sec. 2, Linong Street, Beitou, Taipei City 112, Taiwan; ^2^Department of Physical Medicine and Rehabilitation, School of Medicine, National Defense Medical Center, No. 325, Sec. 2, Chenggong Road, Neihu District, Taipei City 114, Taiwan; ^3^Department of Physical Medicine and Rehabilitation, Tri-Service General Hospital, No. 325, Sec. 2, Chenggong Road, Neihu District, Taipei City 114, Taiwan; ^4^Graduate Institute of Medical Sciences, National Defense Medical Center, No. 325, Sec. 2, Chenggong Road, Neihu District, Taipei City 114, Taiwan; ^5^Department of Nursing, Tri-Service General Hospital, School of Nursing, National Defense Medical Center, No. 325, Sec. 2, Chenggong Road, Neihu District, Taipei City 114, Taiwan; ^6^Department of Physical Medicine and Rehabilitation, Taichung Veterans General Hospital, No. 1650, Sec. 4, Taiwan Boulevard, Xitun District, Taichung City 407, Taiwan

## Abstract

We developed a mobility detection system to analyze pressure changes over time during side-turns in 29 healthy volunteers (17 males and 12 females) with a mean age of 46.1 ± 19.64 years (ranging from 23 to 86 years) in order to determine the effect of gender, age, and BMI on performance during bed postural change. Center of gravity (COG) location, peak pressure of counteraction, and time to reach peak pressure were the main outcomes used to gauge the ability to make a spontaneous side-turn. Men exhibited significantly higher side-turning force (*P* = 0.002) and back-turning force (*P* = 0.002) compared with women. Subjects with BMI ≥27 kg/m^2^ had significantly higher side-turning force (*P* = 0.007) and back-turning force (*P* = 0.007) compared with those with BMI < 27 kg/m^2^. After adjusting for other covariates, age positively correlated with back-turning time (*P* = 0.033) and negatively correlated with side-turning speed (*P* = 0.005), back-turning speed (*P* = 0.014), side-turning force (*P* = 0.010), and back-turning force (*P* = 0.016), respectively. Turning times negatively correlated with time to reach peak pressure (*P* = 0.008). Our system was effective in detecting changes in turning swiftness in the bed-ridden subject.

## 1. Introduction

Determination of body position on a mattress is important for both management and prevention of bedsores in the bed-ridden patient and prevention of muscle atrophy in stroke patients. The development of pressure ulcers is a significant health concern, especially in the elderly population, as pressure ulcers can lead to serious systemic infections [1, 2]. Approximately 60,000 people each year die from complications related to pressure ulcers [3, 4].

Periodic postural change is the key to prevention of deep tissue injury and subsequent pressure ulcer development. However, to our knowledge, there is no objective technology available to evaluate turning ability. In addition, postural training and monitoring of turning motion recovery are needed in the elderly, the stroke patient, and the disabled.

At present, patient body position is measured by visual inspection or by analysis of either a video monitoring system or expensive, high-resolution arrays of pressure sensors [5–7]. Due to high cost and time constraints on nursing care, often a set of accelerometers tethered to the patient is used as an alternative [3, 8–13]. Brown et al. measured postural changes, including lying, sitting, standing, or walking, by attaching wireless accelerometers to the ipsilateral thigh and ankle [8]. These accelerometers monitor a patient's position based on movement and position of the limbs and could potentially measure different levels of mobility that occur during hospitalization. Bagalà et al. proposed a single body-fixed inertial sensor to provide quantitative description of the lie-to-sit-to-stand-to-walk [13]. Wireless accelerometers have also been used to measure turning in bed when attached to the thigh [9]. A wearable alternative, the so-called “Wearable Wireless Identification and Sensing Platform” or WISP, was designed with a single kinematic sensor capable of real-time monitoring to automatically detect bed entry and exit events. Located over the sternum, the WISP method is the preferred method to detect bed entry and exit in order to avoid falls which commonly occur near the bed in hospitals and residential care facilities [12]. To overcome the challenges faced by accelerometer-based monitoring, that is, a low accuracy, inability to recognize both posture and transportation mode simultaneously, and high computational complexity, Zhang and Poslad proposed a new GPS and foot-force sensor method which combines wearable FF sensors with GPS monitoring via a mobile phone [11]. Overall, accelerometry-based wearable motion detectors have been used for classification of posture and movement, estimation of energy expenditure, fall detection, and balance control evaluation [10] but cannot provide information regarding turning ability in bed.

We designed a low-cost mobility detection system not only to monitor spontaneous bed turning but also to assess turning ability in the bed-ridden patient. This mobility detection system combined a sensor mat (using a minimum number of sensors) with data analysis software to monitor turning ability. This system analyzed pressure changes over time during side-turns in 29 healthy subjects. Gravity center location and velocity during movement, time to complete a side-turn, peak counteraction pressure, and time to reach peak pressure (turning swiftness) were the parameters used to gauge the ability to make a spontaneous side-turn in bed.

## 2. Subjects and Methods

### 2.1. Subjects

This study was approved by the Institutional Review Board of Tri-Service General Hospital, National Defense Medical Center. All subjects gave their written informed consent prior to participation in the study.

**Figure 1:**
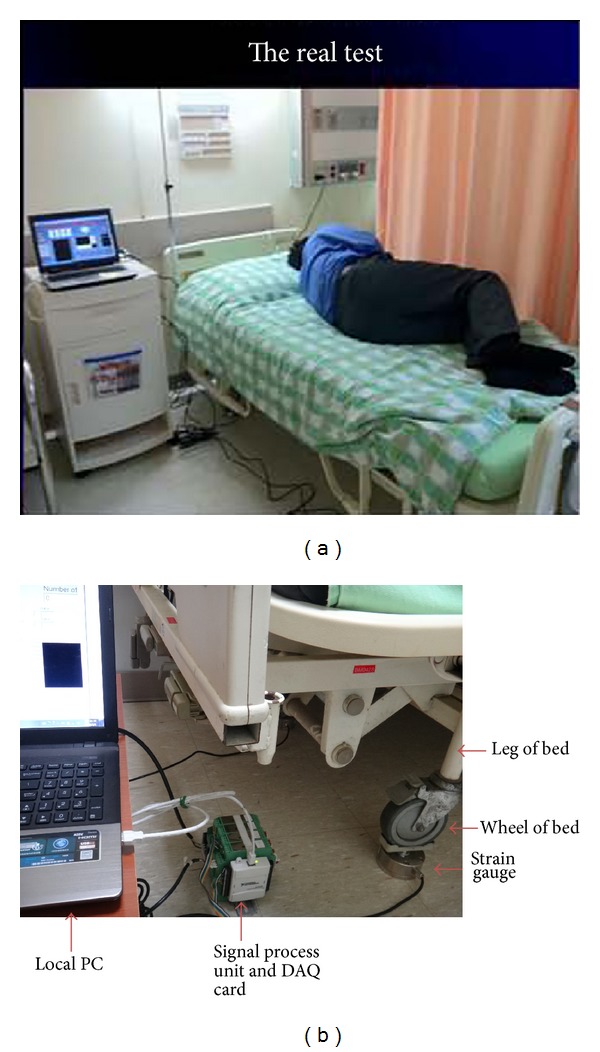
(a) The actual testing apparatus. (b) Close-up showing the sensor attached to the bed leg.

In this study, normal healthy volunteers were asked to lie in the center of the bed and listen to instructions regarding bed turning (Figures 1(a) and 1(b)). Each subject was asked to turn first to their left side, then turn up, wait 10 sec, and then return to lying on their back and wait an additional 10 sec. This task was considered one complete turn and repeated 10 times. After the above task was completed, the same volunteer was asked to turn to the right and then turn up, and this task was repeated 10 times. Thus, for every test, the volunteer performed 20 turns. In order for the subject to recognize when to start and stop each motion, the physician gave clear orders for each turn, such as “turn left,” “turn up,” and so forth.

Data was collected over 20 turns back and forth for each subject. The force generated on the bed surface was recorded and analyzed, as described below.

### 2.2. Mobility Detection System

**Figure 2:**
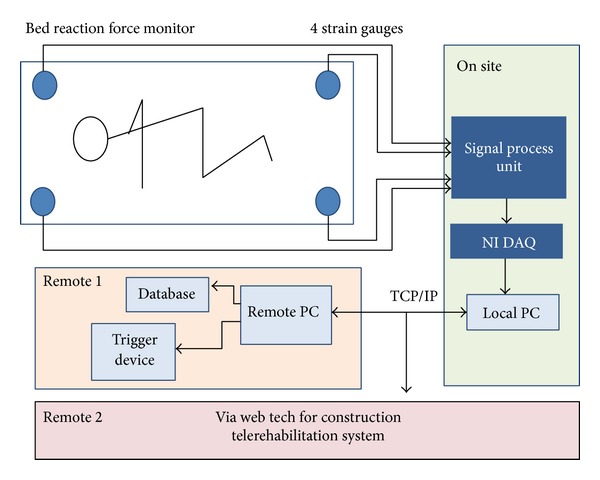
The mobility detection system (patent number M43319-Taiwan).

The mobility detection system was composed of four strain gauges (LFS1CC 150 kg, Delta Transducers Co.) and additional data analysis software (Figure 2). The strain gauges were installed at each of the four legs of a bed (Figure 1(b)). When the subject rolled up onto his or her side, the change in center of pressure was recorded by each of the four strain gauges. We used lever principle to calculate the transposition of force in *XY* plane, as follows:

(1) $$\begin{array}{c} \overset{}{\underset{}{\sum }}Fz=W=W1+W2+W3+W4,\\ \overset{}{\underset{}{\sum }}My:xW=x1W1+x2W2+x3W3+x4W4,\\ \overset{}{\underset{}{\sum }}Mx:yW=y1W1+y2W2+y3W3+y4W4,\\ \text{coF}x≅\frac{My}{Fz};\;\;\text{coF}y≅\frac{Mx}{Fz}, \end{array}$$

**Figure 3:**
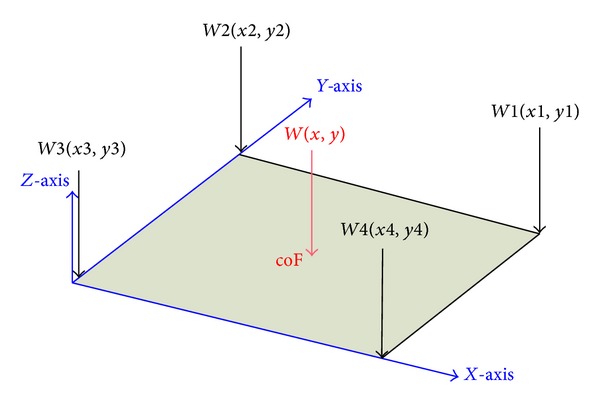
The sum of the changes in pressure readings from the four strain gauges is used to calculate the change in location of the subject on the bed over time (change in the center of pressure or center of force (coF)).

As graphically depicted in Figure 3, the sum of the changes in pressure readings from the four strain gauges was used to calculate the change in location of the subject on the bed over time (change in the center of pressure or center of force (coF)) using the following equation:

(2) $$\begin{array}{c} \Delta \text{coF}=\Delta \left(cx,y\right), \end{array}$$

where

(3) $$\begin{array}{c} cx\left(\text{center of horizontal force}\right)=\frac{\sum aix*Fi}{\sum Fi}\;\left(i=1\;\text{to}\;4\right),\\ cy\left(\text{center of vertical force}\right)=\frac{\sum aiy*Fi}{\sum Fi}\;\left(i=1\;\text{to}\;4\right). \end{array}$$

∑*aix*∗*Fi* refers to the four load cells to withstand the forces generated on the *X*-axis to the total moment, and *aix* is the *x*-coordinate of four load cells, ∑*aiy*∗*Fi* refers to the four load cells to withstand the forces generated on the *Y*-axis to the total moment, and *aiy* is the *y*-coordinate of four load cells.

The analog pressure readings were sent to a signal processing unit and then to the NI-DAQmx8.9 (National Instruments Corp., Austin, TX, USA) which performed analog to digital conversion. The resulting digital signals were input to a PC for data analysis (Figure 2). The data analysis program was written in Labview 2010 (National Instruments Corporation, Austin, TX, USA). The maximum sampling frequency was 100 Hz, and the maximum voltage range was ±10 V. The choice of 100 Hz for the frequency was based on a mean turn time of 5.7 sec. It was felt that 100 Hz was sufficient frequency to monitor the turn time. In addition, if we used 1000 Hz, it would have resulted in the accumulation of an enormous amount of data while monitoring over extended periods such as 24 hour observations. This system was also designed to allow for additional monitoring of multiple beds and remote monitoring, if needed, using the internet.

In summary, this study was designed to monitor coF or center of gravity (COG) changes. To clarify, (i) velocity of COG is equivalent to “turn/back speed,” (ii) peak pressure of counteraction is equivalent to “turn/back force,” and (iii) time to reach peak pressure is equivalent to “turn/back peak time.” Main outcomes of the study were velocity of COG, peak pressure of counteraction, and time to reach peak pressure (turn/back speed, turn/back force, and turn/back peak time). Secondary outcomes included movement distance of COG, side-turn time, and location of COG (TL/BL, turn time/back time, turn/back-weight ratio).

### 2.3. Parameter Definitions

The following terms were used throughout the study.

Turn time (sec) or side-turn time refers to the time to complete the left (or right) side-turn from the back position (resting position); TL (cm) or movement distance of COG refers to gravity distance traveled to complete the left (or right) side-turn; turn speed (cm/sec) or velocity of COG refers to the speed used to complete the left (or right) side-turn and was equal to TL divided by turn time; turn peak time (ds) or time to reach peak pressure refers to turning swiftness when completing a side-turn; turn force (kg·m/s²) or peak pressure of counteraction refers to maximum reaction force expended to complete either the left (or right) side-turn; back time (sec) or side-turn time refers to time to return to one's back from either the left (or right) side-turn; BL (cm) or movement distance of COG refers to distance traveled upon returning to one's back after completing the left (or right) side-turn; back speed (cm/sec) or velocity of COG refers to the speed of turning onto one's back from either the left (or right) side-turn; back peak time (ds) or time to reach peak pressure refers to turning swiftness when completing a back-turn; back force (kg·m/s²) or peak pressure of counteraction refers to maximum reaction force expended to complete the turn onto one's back from either the left (or right) side-turn; turn-weight ratio or location of COG refers to the ratio of turn force to weight; back-weight ratio or location of COG refers to the ratio of back force to weight.

### 2.4. Force Sensor Reliability and Validity

Using a 30 kg standard, each point of the test was repeated ten times, and the test-retest reliability (0.947 for position detected and 0.986 for weight measured) and concurrent validity (0.927 for position detected and 0.924 for weight measured) of the results were tested with regard to location and the weight measured, respectively. Both validity and reliability were found to be above 0.9. According to a systematic review by Wind et al. [14], the criteria of a high test-retest reliability and a high concurrent validity were above 0.8 (*r* > 0.8) and 0.75 (*r* > 0.75), respectively. The force sensor used in this study met both criteria with high validity and reliability.

### 2.5. Statistical Analysis

Continuous variables were summarized using mean ± SD and categorical variables were expressed as frequencies and percentages. The intrarater reliability of parameters that were used to evaluate turning ability was measured by intraclass correlation coefficients (ICCs). The repeated measures analysis of variance (ANOVA) was used to detect the within-subject (side-turning) and between-subject effects (gender, age, and BMI) on various parameters related to turning ability. To investigate the factors associated with turning ability, the generalized estimating equation (GEE) model, with an identity link function, was applied to accommodate the correlated data of repeat measurements within the same subjects. All statistical analyses were performed using SAS software version 9.2 (SAS Institute Inc., Cary, NC). A two-tailed *P* < 0.05 indicated statistical significance.

## 3. Results

### 3.1. Subject Demographics

A total of 29 healthy volunteers, consisting of 17 males (58.6%) and 12 females (41.4%), were enrolled in the study. Their demographics are summarized in Table 1. The mean age of the subjects was 46.1 ± 19.64 years (ranging from 23 to 86 years). Their average body height and body weight were 163.5 ± 6.9 cm and 63.66 ± 12.13 kg, respectively. The average BMI was 23.68 ± 3.49 kg/m^2^ (ranging from 17.15 to 31.55 kg/m^2^).

### 3.2. Intrarater Reliability

For evaluation of intrarater reliability, a separate pilot study of 10 subjects (not involving the 29 subjects used in this study) was conducted and 20 repeat measurements per each parameter (turn time, TL, turn speed, turn peak time, turn force, back time, BL, back speed, back peak time, back force, turn-weight ratio, and back-weight ratio) were taken by one physician (rater) for each subject.

Before the main study began, we wished to investigate reliability of these parameters for measuring turning ability within the same physician (rater). The intrarater reliability of parameters that describe turning ability was quantified using intraclass correlation coefficients (ICCs) [15]. An ICC > 0.75 indicates excellent reliability [16]. The ICCs calculated were as follows: turn time, ICC = 0.609; TL, ICC = 0.929; turn speed, ICC = 0.866; back time, ICC = 0.854; BL, ICC = 0.966; back speed, ICC = 0.942; turn peak time, ICC = 0.571; back peak time, ICC = 0.835; turn force, ICC = 0.993; back force, ICC = 0.998; turn-weight ratio, ICC = 0.983; and back-weight ratio, ICC = 0.996.

### 3.3. Comparison of Parameters Related to Turning Ability

There was no significant difference between left versus right side-turning (Table 2). Men had significantly higher turn force (*P* = 0.002) and back force (*P* = 0.002) when compared with women (Table 3). Healthy volunteers with BMI ≥ 27 kg/m^2^had significantly higher turn force (*P* = 0.007) and back force (*P* = 0.007) when compared with those who had BMI < 27 kg/m^2^ (Table 4). Subjects ≥ 65 years of age took significantly longer time to reach peak swiftness (turn peak time) (*P* = 0.025) compared to those < 65 years old (Table 5).

### 3.4. Factors Associated with Parameters of Turning Ability: The GEE Model (Table 6)

The GEE models included all variables in the multivariate analysis. After adjusting for other covariates, factors associated with parameters related to turning ability were as follows: (i) men had higher turn force (*P* = 0.020) and back force (*P* = 0.041) than women; (ii) BMI positively correlated with higher TL (*P* = 0.047), turn force (*P* < 0.001), and back force (*P* < 0.001), respectively; (iii) age positively correlated with back time (*P* = 0.033) and negatively correlated with turn speed (*P* = 0.005), back speed (*P* = 0.014), turn force (*P* = 0.010), and back force (*P* = 0.016), respectively; (iv) turning times negatively correlated with turn peak time (turning swiftness) (*P* = 0.008).

## 4. Discussion

Our mobility detection system was the first to quantify turning ability in the elderly subject, underscoring the originality of this research. In addition, we found that men exhibited significantly higher side-turning force and back-turning force compared with women. Healthy volunteers with BMI ≥ 27 kg/m^2^ had significantly higher side-turning force (and back-turning force) compared with those who had BMI < 27 kg/m^2^. Interestingly, subjects ≥ 65 years old took significantly longer to reach peak turning swiftness compared with those subjects < 65 years old. Multivariate analysis showed that age positively correlated with back-turning time and negatively correlated with side-turning speed, back-turning speed, side-turning force, and back-turning force.

Slow turning speed in the elderly may arise from several causes including loss of skeletal muscle mass and force [17] as well as loss of balance with increasing age [18]. Proprioceptive acuity also degrades over time in the older patient and has been directly correlated with falls and reduced functional independence [19]. It may also be a contributing factor in the reduction of turning speed with aging. Although such factors are understandable, this study is the first to present quantitative data on the decline in turning ability in the elderly. These novel findings can also be used to lay a foundation for monitoring those patients who have reduced turning ability due to long-term lack of activity, such as chronic bed-ridden patients, and can be further correlated with functional scales. In addition, they underscore the need for aggressive physical therapy, especially side-turning in the bed-ridden patient, as it has been shown that regular physical activity is an important strategy in attenuating age-related decay of muscle structure and function [17].

The ability to complete a side-turn is an important activity in bed-ridden patients [20], as a good side-turn can reduce the likelihood of developing bedsores [1], reverse deconditioning [21], and improve mobility [22]. Ability to complete a side-turn requires motor coordination involving balance, proprioception, and cerebellar function [23–28]. During infancy, this side-turning function is accomplished as an important first step in learning to walk without support; that is, the developmental stages include turning over, sitting, crawling, standing, and, finally, walking [26]. At approximately 4–6 months of age, the infant progresses from the ability to turn over to the ability to stand up, an action requiring coordination of nerve function, muscle strength, and bone support. Unfortunately, when this ability is impaired in bed-ridden patients such as stroke victims, or patients in the intensive care unit (ICU), it represents a decline in basic activity and functioning. However, no quantifiable clinical turning tool has yet been developed to monitor this decline in function.

The prevention of bedsores through side-turns is a very important and relevant topic. Several factors are involved in the development of bedsores and can be divided into intrinsic versus extrinsic factors. Intrinsic factors include nutrition and skin humidity, while extrinsic factors include direct skin pressure, shear forces, and/or friction [29]. Generally, direct pressure has the greatest impact on pressure sore development [4, 30, 31]. Therefore, it is clinically important to stand the patient up every two hours to prevent or treat pressure ulcers, but currently there is no particular requirement for side-turning that is quantifiable. Our system allows for such monitoring as well as monitoring the frequency of side-turns.

Once bedsores develop, they represent an acute health condition that results in increased cost and suffering over months or even years. Effective ulcer prevention and early detection is, therefore, necessary. Strong motivation for a computerized mobility detection system arises from the growing shortage of trained health care providers [32] and the ever-increasing cost of health care. One particular multimodal approach to monitoring sleeping posture, using a pressure sensor array and video camera as complementary modalities, has been proposed [7, 33]. Unfortunately, the use of cameras in nursing units entails certain privacy issues which must be considered and camera surveillance also involves nursing time which tends to drain an already encumbered health care system [7, 32].

### 4.1. Study Limitations

Our study had several limitations. Participants were all healthy volunteers with a mean age of 46.1 ± 19.64 yrs (ranging from 23 to 86 yrs). Thus, no patients at risk for developing bed sores (such as stroke, spinal cord injury, or ICU patients) were included in the study. Further validation of our method in patients with (or at high risk for) impaired side-turning on beds is required. Overnight monitoring of older healthy individuals and patients using our mobility detection system is also required for complete assessment. Reliability of the mobility detection system should also be examined by video monitoring system. Since this is the first study of its kind to analyze turning swiftness, further clinical trials examining a larger aging population are required to confirm our results. In particular, turn angle, turn angle speed, and other related measures should be considered in future clinical trials since they are less dependent on the height or width of the participants.

## 5. Conclusion

Our mobility detection system has proven to be effective in detecting changes in turning swiftness on beds. It is effective and easy to use. However, further validation with patients at high risk for impaired side-turning on beds is required.

## Figures and Tables

**Table 1:** Subject demographics.

Variables	*N* = 29
Age	
Mean ± SD	46.1 ± 19.64
Range	23–86
Gender, *n* (%)	
Male	17 (58.6)
Female	12 (41.4)
Height (cm)	
Mean ± SD	163.5 ± 6.9
Range	150–181
Weight(cm)	
Mean ± SD	63.66 ± 12.13
Range	45–88
BMI (kg/m^2^)	
Mean ± SD	23.68 ± 3.49
Range	17.15–31.55

BMI: body mass index.

**Table 2:** Turning ability parameters.

	Left side-turn		Right side-turn		
	*n* ^a^	Mean ± SD	*n*	Mean ± SD	*P*-value^†^
TURN Time (sec)	290	5.71 ± 0.75	290	5.75 ± 1.27	0.797
TL (cm)	290	24.35 ± 8.48	290	24.46 ± 8.68	0.871
TURN Speed (cm/sec)	290	4.31 ± 1.57	290	4.37 ± 1.72	0.551
TURN Peak Time (ds)	290	8.44 ± 7.65	290	8.06 ± 6.99	0.750
TURN Force (kg·m/s²)	290	64.49 ± 13.10	290	65.14 ± 12.87	0.448
BACK Time (sec)	290	5.17 ± 1.39	290	5.29 ± 1.87	0.533
BL (cm)	290	24.66 ± 8.46	290	24.64 ± 8.77	0.974
BACK Speed (cm/sec)	290	4.89 ± 1.72	290	4.84 ± 1.94	0.754
BACK Peak Time (ds)	290	11.14 ± 13.76	290	10.90 ± 6.38	0.862
BACK Force (kg·m/s²)	290	65.03 ± 13.50	290	65.87 ± 13.25	0.183
TURN Weight ratio	290	1.015 ± 0.103	290	1.031 ± 0.139	0.320
BACK Weight ratio	290	1.024 ± 0.115	290	1.041 ± 0.132	0.129

^a^Numbers of measurements; ^†^determined by repeated measures ANOVA (within-subject effect).

TURN Time (sec): time to complete left (or right) side-turn from the back position (resting position).

TL (cm): distance traveled to complete left (or right) side-turn.

TURN Speed (cm/sec): speed (distance/time) used to complete left (or right) side-turn.

TURN Peak Time (ds): time to reach peak pressure (turning swiftness) when completing side-turn.

TURN Force (kg·m/s²): maximum reaction force expended to complete either left (or right) side-turn.

BACK Time (sec): time to return to one's back from either left (or right) side-turn.

BL (cm): distance traveled upon returning to one's back after completing left (or right) side-turn.

BACK Speed (cm/sec): speed (distance/time) of turning unto one's back from either left (or right) side-turn.

BACK Peak Time (ds): time to reach peak pressure (turning swiftness) when completing back-turn.

BACK Force (kg·m/s²): maximum reaction force expended to complete the turn unto one's back from either left (or right) side-turn.

TURN Weight ratio: ratio of TURN force to weight.

BACK Weight ratio: ratio of BACK force to weight.

**Table 3:** Gender differences in turning ability.

	Men		Women		
	*n* ^a^	Mean ± SD	*n*	Mean ± SD	*P* value^†^
Turn time (sec)	340	5.60 ± 0.79	240	5.91 ± 1.29	0.226
TL (cm)	340	24.12 ± 8.09	240	24.81 ± 9.22	0.827
Turn speed (cm/sec)	340	4.33 ± 1.41	240	4.36 ± 1.93	0.965
Turn peak time (ds)	340	7.89 ± 6.83	240	8.77 ± 7.96	0.482
Turn force (kg·m/s²)	340	70.80 ± 11.89	240	56.34 ± 9.18	0.002*
Back time (sec)	340	5.23 ± 1.98	240	5.22 ± 0.99	0.947
BL (cm)	340	24.35 ± 8.14	240	25.07 ± 9.24	0.823
Back speed (cm/sec)	340	4.81 ± 1.69	240	4.95 ± 2.02	0.834
Back peak time (ds)	340	11.59 ± 13.09	240	10.21 ± 5.83	0.331
Back force (kg·m/s²)	340	71.42 ± 12.74	240	56.99 ± 8.98	0.002*
Turn-weight ratio	340	1.015 ± 0.108	240	1.035 ± 0.139	0.642
Back-weight ratio	340	1.023 ± 0.121	240	1.047 ± 0.127	0.595

^a^Numbers of measurements; ^†^determined by repeated measures ANOVA (within-subject effect).

*: *P* < 0.05 indicated statistical significance.

**Table 4:** Association between BMI and turning ability of healthy volunteers.

	BMI < 27 kg/m^2^		BMI ≥ 27 kg/m^2^		
	*n* ^a^	Mean ± SD	*n*	Mean ± SD	*P* value^†^
Turn time (sec)	440	5.71 ± 1.08	140	5.80 ± 0.90	0.782
TL (cm)	440	23.30 ± 7.85	140	27.89 ± 9.77	0.203
Turn speed (cm/sec)	440	4.16 ± 1.57	140	4.89 ± 1.76	0.292
Turn peak time (ds)	440	8.16 ± 7.06	140	8.52 ± 8.12	0.802
Turn force (kg·m/s²)	440	61.30 ± 11.05	140	75.86 ± 12.40	0.007*
Back time (sec)	440	5.14 ± 1.27	140	5.51 ± 2.47	0.160
BL (cm)	440	23.52 ± 7.87	140	28.19 ± 9.82	0.202
Back speed (cm/sec)	440	4.70 ± 1.71	140	5.39 ± 2.10	0.366
Back peak time (ds)	440	10.47 ± 11.69	140	12.75 ± 6.57	0.161
Back force (kg·m/s²)	440	61.84 ± 11.34	140	76.80 ± 12.96	0.007*
Turn-weight ratio	440	1.044 ± 0.115	140	0.957 ± 0.123	0.077
Back-weight ratio	440	1.053 ± 0.115	140	0.969 ± 0.131	0.096

^a^Numbers of measurements; ^†^determined by repeated measures ANOVA (within-subject effect).

*: *P* < 0.05 indicated statistical significance.

**Table 5:** Association between age and turning ability of healthy volunteers.

	Age < 65 year		Age ≥ 65 year		*P* value^†^
	*n* ^a^	Mean ± SD	*n*	Mean ± SD	
Turn time (sec)	460	5.65 ± 1.03	120	6.04 ± 1.04	0.221
TL (cm)	460	25.26 ± 8.80	120	21.13 ± 6.75	0.280
Turn speed (cm/sec)	460	4.57 ± 1.72	120	3.46 ± 0.86	0.127
Turn peak time (ds)	460	7.57 ± 6.88	120	10.84 ± 8.37	0.025*
Turn force (kg·m/s²)	460	66.17 ± 12.73	120	59.64 ± 12.65	0.276
Back time (sec)	460	5.13 ± 1.25	120	5.60 ± 2.64	0.089
BL (cm)	460	25.49 ± 8.88	120	21.40 ± 6.57	0.293
Back speed (cm/sec)	460	5.09 ± 1.88	120	4.00 ± 1.36	0.167
Back peak time (ds)	460	11.14 ± 11.53	120	10.56 ± 6.78	0.742
Back force (kg·m/s²)	460	66.69 ± 13.14	120	60.71 ± 13.25	0.334
Turn-weight ratio	460	1.040 ± 0.108	120	0.958 ± 0.149	0.114
Back-weight ratio	460	1.048 ± 0.109	120	0.975 ± 0.158	0.173

^a^Numbers of measurements; ^†^determined by repeated measures ANOVA (within-subject effect).

*: *P* < 0.05 indicated statistical significance.

**Table 6:** Generalized predictors affecting turning ability.

Variables	Univariate		Multivariate	
	Beta (95% CI)	*P* value	Beta (95% CI)	*P* value
Turn time (sec)				
Gender (men versus women)	−0.310 (−0.819, 0.198)	0.232	−0.414 (−0.842, 0.014)	0.058
BMI (per kg/m^2^)	0.004 (−0.063, 0.071)	0.904	0.028 (−0.022, 0.078)	0.267
Age (per year)	0.010 (−0.002, 0.023)	0.106	0.010 (−0.002, 0.022)	0.114
Side-turn (right versus left)	0.040 (−0.255, 0.334)	0.792	0.040 (−0.255, 0.334)	0.792
Times	−0.007 (−0.039, 0.026)	0.685	−0.007 (−0.039, 0.026)	0.685
TL (cm)				
Gender (men versus women)	−0.697 (−6.788, 5.395)	0.823	−2.990 (−8.883, 2.903)	0.320
BMI (per kg/m^2^)	0.377 (−0.323, 1.076)	0.291	0.673 (0.010, 1.336)	0.047*
Age (per year)	−0.125 (−0.262, 0.011)	0.072	−0.137 (−0.277, 0.003)	0.056
Side-turn (right versus left)	0.116 (−1.250, 1.483)	0.868	0.116 (−1.250, 1.483)	0.868
Times	0.089 (−0.008, 0.185)	0.071	0.089 (−0.008, 0.185)	0.071
Turn speed (cm/sec)				
Gender (men versus women)	−0.027 (−1.234, 1.181)	0.966	−0.377 (−1.577, 0.823)	0.538
BMI (per 1 kg/m^2^)	0.058 (−0.075, 0.191)	0.391	0.104 (−0.017, 0.226)	0.093
Age (per 1 year)	−0.031 (−0.052, −0.009)	0.005	−0.032 (−0.055, −0.010)	0.005*
Side-turn (right versus left)	0.059 (−0.130, 0.248)	0.539	0.059 (−0.130, 0.248)	0.539
Times	0.021 (0.000, 0.041)	0.050	0.021 (0.000, 0.041)	0.050
TURN Peak Time (ds)				
Gender (men versus women)	−0.878 (−3.271, 1.515)	0.472	−1.235 (−3.747, 1.277)	0.335
BMI (per kg/m^2^)	0.036 (−0.308, 0.381)	0.836	0.095 (−0.267, 0.457)	0.606
Age (per year)	0.053 (−0.004, 0.110)	0.066	0.052 (−0.003, 0.107)	0.066
Side-turn (right versus left)	−0.382 (−2.664, 1.901)	0.743	−0.382 (−2.664, 1.901)	0.743
Times	−0.185 (−0.321, −0.049)	0.008	−0.185 (−0.321, −0.049)	0.008*
TURN Force (kg·m/s²)				
Gender (men versus women)	14.465 (7.121, 21.808)	0.000	6.708 (1.038, 12.378)	0.020*
BMI (per kg/m^2^)	2.627 (1.778, 3.476)	<0.0001	2.237 (1.432, 3.042)	<0.0001*
Age (per year)	−0.117 (−0.347, 0.114)	0.320	−0.160 (−0.282, −0.039)	0.010*
Side-turn (right versus left)	0.651 (−0.977, 2.279)	0.433	0.651 (−0.977, 2.279)	0.433
Times	−0.048 (−0.108, 0.012)	0.119	−0.048 (−0.108, 0.012)	0.119
BACK Time (sec)				
Gender (men versus women)	0.016 (−0.411, 0.442)	0.943	−0.113 (−0.442, 0.217)	0.503
BMI (per kg/m^2^)	0.034 (−0.030, 0.098)	0.303	0.035 (−0.014, 0.084)	0.158
Age (per year)	0.012 (0.001, 0.023)	0.032	0.011 (0.001, 0.022)	0.033*
Side-turn (right versus left)	0.121 (−0.247, 0.488)	0.520	0.121 (−0.247, 0.488)	0.520
Times	−0.006 (−0.055, 0.042)	0.803	−0.006 (−0.055, 0.042)	0.803
BL (cm)				
Gender (men versus women)	−0.722 (−6.905, 5.462)	0.819	−3.025 (−9.116, 3.065)	0.330
BMI (per kg/m^2^)	0.377 (−0.336, 1.089)	0.300	0.676 (−0.016, 1.368)	0.055
Age (per year)	−0.127 (−0.265, 0.012)	0.073	−0.138 (−0.282, 0.005)	0.058
Side-turn (right versus left)	−0.022 (−1.320, 1.276)	0.973	−0.022 (−1.320, 1.276)	0.973
Times	0.039 (−0.050, 0.128)	0.389	0.039 (−0.050, 0.128)	0.389
BACK Speed (cm/sec)				
Gender (men versus women)	−0.139 (−1.426, 1.148)	0.833	−0.500 (−1.758, 0.757)	0.436
BMI (per kg/m^2^)	0.052 (−0.096, 0.199)	0.492	0.108 (−0.032, 0.248)	0.131
Age (per year)	−0.032 (−0.059, −0.006)	0.016	−0.034 (−0.061, −0.007)	0.014*
Turn side (right versus left)	−0.053 (−0.376, 0.270)	0.747	−0.053 (−0.376, 0.270)	0.747
Times	0.005 (−0.016, 0.026)	0.631	0.005 (−0.016, 0.026)	0.631
BACK Peak Time (ds)				
Gender (men versus women)	1.389 (−1.105, 3.882)	0.275	0.473 (−2.984, 3.929)	0.789
BMI (per kg/m^2^)	0.289 (−0.047, 0.625)	0.092	0.264 (−0.214, 0.741)	0.279
Age (per year)	−0.010 (−0.065, 0.045)	0.718	−0.015 (−0.066, 0.036)	0.559
Side-turn (right versus left)	−0.231 (−2.757, 2.296)	0.858	−0.231 (−2.757, 2.296)	0.858
Times	−0.007 (−0.331, 0.316)	0.965	−0.007 (−0.331, 0.316)	0.965
BACK Force (kg·m/s²)				
Gender (men versus women)	14.430 (6.843, 22.018)	0.0002	6.380 (0.262, 12.497)	0.041*
BMI (per kg/m^2^)	2.687 (1.819, 3.555)	<0.0001	2.321 (1.467, 3.175)	<0.0001*
Age (per year)	−0.116 (−0.357, 0.125)	0.344	−0.161 (−0.292, −0.030)	0.016*
Side-turn (right versus left)	0.847 (−0.347, 2.042)	0.165	0.847 (−0.347, 2.042)	0.165
Times	0.032 (−0.065, 0.128)	0.519	0.032 (−0.065, 0.128)	0.519
TURN Weight ratio				
Gender (men versus women)	−0.020 (−0.105, 0.064)	0.637	0.015 (−0.065, 0.096)	0.708
BMI (per kg/m^2^)	−0.009 (−0.021, 0.002)	0.112	−0.010 (−0.022, 0.001)	0.082
Age (per year)	−0.001 (−0.003, 0.001)	0.440	−0.001 (−0.003, 0.001)	0.540
Side-turn (right versus left)	0.015 (−0.014, 0.044)	0.302	0.015 (−0.014, 0.044)	0.302
Times	−0.001 (−0.002, 0.000)	0.163	−0.001 (−0.002, 0.000)	0.163
BACK Weight ratio				
Gender (men versus women)	−0.024 (−0.108, 0.061)	0.5805	0.009 (−0.077, 0.094)	0.844
BMI (per kg/m^2^)	−0.009 (−0.021, 0.003)	0.1285	−0.009 (−0.021, 0.003)	0.127
Age (per year)	−0.001 (−0.003, 0.001)	0.4668	−0.001 (−0.003, 0.002)	0.550
Side-turn (right versus left)	0.017 (−0.004, 0.037)	0.1114	0.017 (−0.004, 0.037)	0.111
Times	0.000 (−0.001, 0.002)	0.5404	0.000 (−0.001, 0.002)	0.540

*: *P* < 0.05 indicated statistical significance.
